# Genetic variations in *AURORA* cell cycle kinases are associated with glioblastoma multiforme

**DOI:** 10.1038/s41598-021-96935-y

**Published:** 2021-08-31

**Authors:** Aner Mesic, Marija Rogar, Petra Hudler, Nurija Bilalovic, Izet Eminovic, Radovan Komel

**Affiliations:** 1 Department of Biology, Faculty of Science, University of Sarajevo, Zmaja od Bosne 33-35, 71000 Sarajevo, Bosnia and Herzegovina; 2 Medical Centre for Molecular Biology, Institute of Biochemistry and Molecular Genetics, Faculty of Medicine, University of Ljubljana, Vrazov trg 2, 1000 Ljubljana, Slovenia; 3 Clinical Pathology and Cytology, University Clinical Centre Sarajevo, Bolnička 25, 71000 Sarajevo, Bosnia and Herzegovina

**Keywords:** Cancer genetics, CNS cancer, Biomarkers, Molecular medicine, Oncology, Risk factors

## Abstract

Glioblastoma multiforme (GBM) is the most frequent type of primary astrocytomas. We examined the association between single nucleotide polymorphisms (SNPs) in Aurora kinase A (*AURKA*), Aurora kinase B (*AURKB*), Aurora kinase C (*AURKC*) and Polo-like kinase 1 (*PLK1*) mitotic checkpoint genes and GBM risk by qPCR genotyping. In silico analysis was performed to evaluate effects of polymorphic biological sequences on protein binding motifs. Chi-square and Fisher statistics revealed a significant difference in genotypes frequencies between GBM patients and controls for *AURKB* rs2289590 variant (*p* = 0.038). Association with decreased GBM risk was demonstrated for *AURKB* rs2289590 AC genotype (OR = 0.54; 95% CI = 0.33–0.88; *p* = 0.015). Furthermore, *AURKC* rs11084490 CG genotype was associated with lower GBM risk (OR = 0.57; 95% CI = 0.34–0.95; *p* = 0.031). Bioinformatic analysis of rs2289590 polymorphic region identified additional binding site for the Yin-Yang 1 (YY1) transcription factor in the presence of C allele. Our results indicated that rs2289590 in *AURKB* and rs11084490 in *AURKC* were associated with a reduced GBM risk. The present study was performed on a less numerous but ethnically homogeneous population. Hence, future investigations in larger and multiethnic groups are needed to strengthen these results.

## Introduction

Glioblastoma multiforme (GBM) represents the most common and lethal form of primary brain tumor with an annual incidence of 5.26 per 100,000 people^1,2^ and it stands for more than 60% of all brain tumors in adults^3^. Although a significant number of modern therapies against GBM is available, it is still a deadly disease with a poor prognosis^4^. Precise chromosomal segregation in dividing cancer cells as well as disturbances during the spindle assembly checkpoint can contribute to malignant transformation^5^. Genetic modifications in mitotic genes could increase sensitivity to neoplastic transformation through alterations of gene expression profiles^6,7^. Aurora kinases are members of serine-threonine kinases family which are of great importance for the cell cycle control^8^. Aurora kinase A (*AURKA*) is involved in proper functioning of a few oncogenic signaling processes such as mitotic entry, spindle assembly, centrosome functioning, chromosome alignment and/or segregation and cytokinesis^9–11^. Aurora kinase B (*AURKB*) is a component of chromosomal passenger complex and mediates in chromatin modification, spindle checkpoint regulation, cytokinesis and correct kinetochore/microtubule attachment^9,12^. Aurora kinase C (*AURKC*) is also a member of chromosomal passenger complex which takes part in mitotic events such as accurate centrosome functioning^13^, and is required to regulate chromosome segregation during meiosis I^14^. Polo-like kinase 1 (*PLK1*) is engaged in several cellular processes including centrosome maturation, mitotic checkpoint activation and spindle assembly, kinetochore/microtubule binding, cytokinesis and cellular proliferation^15–17^. *PLK1* overexpression is proved to be associated with poor prognosis in several cancer entities^18^. Moreover, it has been demonstrated that polymorphisms in *PLK1* affect its expression, thus possess the ability to potentially influence the risk of disease onset and progression^18^.

In our case–control study, we evaluated the impact of single nucleotide polymorphisms rs1047972, rs2273535, rs8173 and rs911160 (*AURKA*), rs2289590 and rs2241909 (*AURKB*), rs11084490 and rs758099 (*AURKC*) and rs42873 (*PLK1*) in mitotic checkpoint genes on glioblastoma multiforme development in Bosnia and Herzegovina population. Using bioinformatic analysis of genetic variants, we estimated the impact of the polymorphic DNA sequences in introns and untranslated regions (UTRs) within *AURKA*, *AURKB*, *AURKC* and *PLK1* genes on transcription factors binding sites.

## Methods

### Design of the study and study groups

Our study group consisted of 129 patients with diagnosed glioblastoma multiforme (GBM) at the Clinical Pathology and Cytology at the University Clinical Center Sarajevo, Bosnia and Herzegovina. Of that, 68 were men and 61 women, with a mean age of 58 years at the moment of diagnosis (data were missing in 4 cases) (Table 1). The formalin fixed paraffin embedded (FFPE) cancer tissue sections were collected in the course of surgical procedures. Written informed consents, which allow the use of samples in this study, were obtained from all patients prior to the surgery. On the other side, 203 healthy blood donors (ethnicity matched to the cases), upon regular medical examinations, were randomly selected and signed up as a control group for the present study. Control samples had no history of neoplastic formation, were not related to the patients and/or to each other. Three milliliters of blood were taken from each control individual and kept at − 80 °C. An informed written consent was obtained from the participants, with personal and medical information being enciphered in order to ensure maximum anonymity in compliance with the World Medical Association’s Declaration of Helsinki. This study was approved by the University Clinical Centre Sarajevo Ethical Committee (No. 0302-36765).

**Table 1:** General information for glioblastoma multiforme patients.

Variable	GBM patients	
	N	N (%)
Total sample	129	
**Gender**		
Female	61	(47.3)
Male	68	(52.7)
**Age at diagnosis (years)**^**a**^		
< 58	56	(44.8)
≥ 58	69	(55.2)
Mean	58	
Range	19–81	

*GBM* glioblastoma multiforme. ^a^Data were missing in 4 cases.

### DNA extraction

Genomic DNA from glioblastoma multiforme (GBM) formalin fixed paraffin embedded tissues was extracted using the Chemagic FFPE DNA Kit special (PerkinElmer Inc., Waltham, MA, USA). DNA washing and elution was performed on Chemagic Magnetic Separation Module I robot (PerkinElmer Inc., Waltham, MA, USA), following manufacturer’s recommendations. All sample transfers were conducted with the four-eye principle to avoid mixing errors of the samples. DNA from peripheral blood lymphocytes (controls) was isolated using the Promega™ Wizard™ Genomic DNA Purification Kit Protocol (Promega Corp., Fitchburg, WI, USA) in concordance with the manufacturer’s instructions. The qualitative/quantitative analysis of the extracted DNA was performed on the DropSense96 photometer (Trinean, Gentbrugge, Belgium) and Synergy™ 2 Multi Mode Reader (BioTek, Inc., Winooski, VT, USA).

### SNP selection

**Figure 1:**
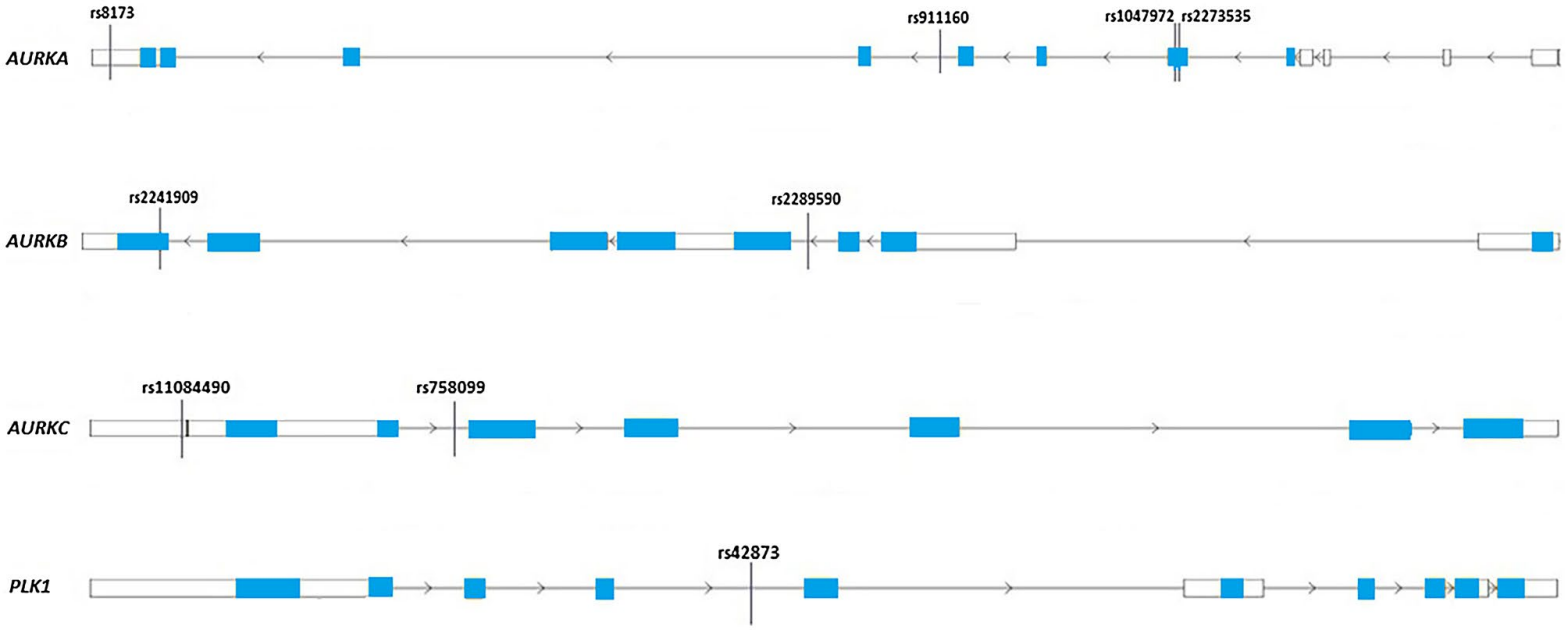
The positions of rs1047972, rs2273535, rs8173 and rs911160 (*AURKA*), rs2289590 and rs2241909 (*AURKB*), rs11084490 and rs758099 (*AURKC*) and rs42873 (*PLK1*) genetic variants within mitotic checkpoint genes. White boxes represent untranslated regions (UTRs). Blue boxes refer to protein coding regions (exons). The black lines connecting the boxes indicate non-coding regions (introns). The gene structures were downloaded from the Research Collaboratory for Structural Bioinformatics (RCSB) Protein Data Bank (PDB), GRCh38 Genome Assembly.

**Figure 2:**
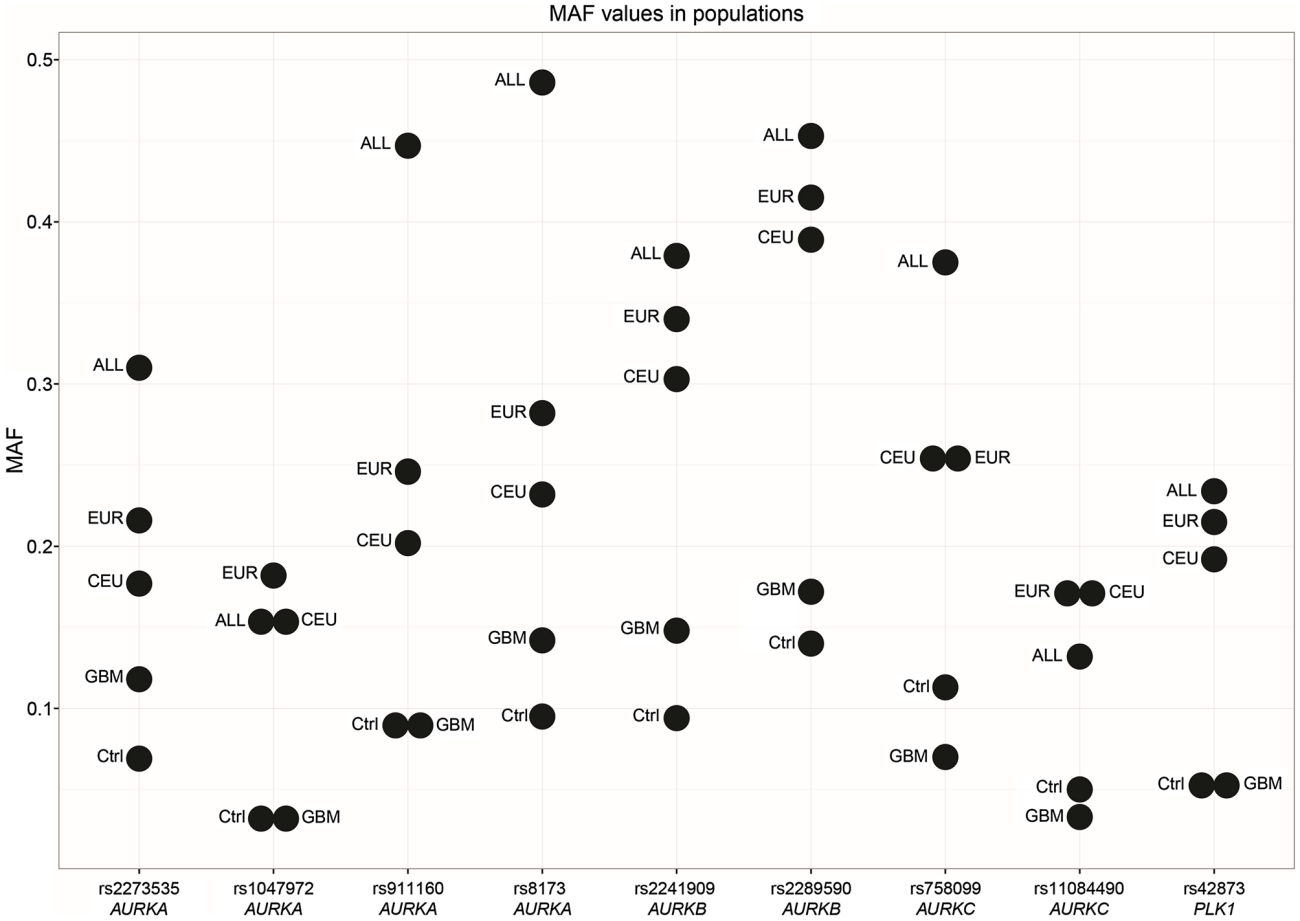
Minor allele frequencies (MAFs) for polymorphisms rs1047972, rs2273535, rs8173 and rs911160 (*AURKA*), rs2289590 and rs2241909 (*AURKB*), rs11084490 and rs758099 (*AURKC*) and rs42873 (*PLK1*) in various populations. *ALL* all individuals from 1000 Genome Project Phase 3 release, *Ctrl* studied control population, *CEU* Utah residents with Northern and Western European ancestry, *EUR* European population, *GBM* studied glioblastoma multiforme group, *MAF* minor allele frequency.

In total, nine single nucleotide polymorphisms (SNPs) in segregation genes, precisely rs1047972, rs2273535, rs8173 and rs911160 (*AURKA*), rs2289590 and rs2241909 (*AURKB*), rs11084490 and rs758099 (*AURKC*) and rs42873 (*PLK1*) were chosen. The locations of the selected variants in mitotic genes are shown in Fig. 1, whereby gene structures were obtained from the Research Collaboratory for Structural Bioinformatics (RCSB) Protein Data Bank (PDB)^19^. The parameters described below are used for the selection of the genetic variants: (1) previously established association related to certain tumors; (2) minor allele frequency (MAF) fewer or equal to 10% in the population of Utah residents with Northern and Western European ancestry (CEU) as highlighted by the Phase 3 1000 Genomes; and (3) tagging single nucleotide polymorphisms (tagSNPs) status, which was computed in silico using LD Tag SNP Selection (tagSNP) (https://snpinfo.niehs.nih.gov)^20^. To predict tagSNPs status, following parameters were used: (a) 1 kb of the upstream–downstream sequences from gene; (b) linkage disequilibrium (LD) lower threshold of 0.8; and (c) minor allele frequency range from 0.05 to 0.5 for the CEU subpopulation (Table 2; Fig. 2).

**Table 2:** Basic characteristics of the studied genetic variants.

SNP	Variant type	Gene	Base change	NCBI assembly location (Build GRCh38)^a^	TaqMan SNP assay ID	MAF^b^				
						GBM patients	Control group	ALL	EUR	CEU
rs1047972	Missense	*AURKA*	C/T	Chr.20:56386407	AHX1IRW	0.162	0.146	0.150	0.182	0.157
rs2273535	Missense	*AURKA*	A/T	Chr.20:56386485	C_25623289_10	0.299	0.238	0.310	0.216	0.177
rs8173	3′ UTR	*AURKA*	G/C	Chr.20:56369735	C_8947675_10	0.354	0.305	0.486	0.282	0.232
rs911160	Intron	*AURKA*	G/C	Chr.20:56382507	C_8947670_10	0.300	0.276	0.447	0.246	0.202
rs2289590	Intron	*AURKB*	C/A	Chr.17:8207446	C_15770418_10	0.375	0.415	0.453	0.415	0.389
rs2241909	Synonymous	*AURKB*	A/G	Chr.17:8205021	C_22272900_10	0.332	0.332	0.379	0.340	0.303
rs11084490	5′ UTR	*AURKC*	C/G	Chr.19:57231104	C_27847620_10	0.152	0.223	0.132	0.165	0.177
rs758099	Intron	*AURKC*	C/T	Chr.19:57231966	C_2581008_1_	0.162	0.302	0.375	0.255	0.253
rs42873	Intron	*PLK1*	G/C	Chr.16:23683411	C_2392140_10	0.354	0.208	0.234	0.215	0.192

*ALL* all phase 3 individuals, *CEU* Utah residents with Northern and Western European ancestry, *EUR* European population, *GBM* glioblastoma multiforme, *MAF* minor allele frequency, *SNP* single nucleotide polymorphism, *UTR* untranslated region. ^a^https://www.lifetechnologies.com. ^b^MAFs extracted from 1000 Genomes Project Phase 3.

### Genotyping of SNPs

Genotyping of the studied variants was performed using TaqMan SNP genotyping assays (Applied Biosystems, Foster City, CA), whose ID numbers are shown in Table 2. The polymerase chain reaction (PCR) mixtures (5 µl for GBM samples and 10 µl for the control samples) consisted of 20X TaqMan^®^ assay along with 2X Master Mix (Applied Biosystems, Foster City, CA), and 20 nanograms of genomic DNA. PCR profile was conducted following manufacturer’s recommendations, hence initial denaturation at 95 °C for 10 min, 45 cycles at 92 °C for 15 s and 60 °C for 90 s, using the ViiA 7 Real Time PCR System (Applied Biosystems, Foster City, CA). At least two negative controls were included in each plate. The results of the PCR reaction were analyzed using TaqMan^®^ Genotyper Software (Applied Biosystems, Foster City, CA, USA).

### Statistical analysis

The genotype frequencies of the polymorphisms, for both case and control populations were tested for Hardy–Weinberg equilibrium (HWE) using Michael H. Court’s online HWE calculator (http://www.tufts.edu)^21^. Significance of the differences in genotype frequencies between GBM patients and controls was determined by use of the Chi-square test or Fisher’s exact test. Multinomial logistic regression was used to test the association between investigated genetic variants and the GBM risk. In this regard, odds ratio (OR) with 95% confidence interval (CI) were calculated to evaluate the relative risk. Statistical analyses were performed using SPSS 20.0 software package (SPSS, Chicago, IL, USA). *P* ≤ 0.05 was chosen as a threshold significance value. Minor allele frequency (MAF) plot was created in R^22^ using ggplot2 R package^23^.

### Analysis of haplotypes

To determine the haplotype block structure and perform haplotype analysis, which included corrections for multiple comparisons by 10,000 permutations, Haploview software, version 4.2^24^ and SNP tools V1.80 (MS Windows, Microsoft Excel) were used. In order to create the haplotype block, solid spine of the linkage disequilibrium (LD) algorithm with a minimum Lewontin’s D′ value of 0.8 was chosen.

### In silico analysis of polymorphisms

Effects of the polymorphic DNA sequences [polymorphisms in non-coding and untranslated regions (UTRs)] on transcription factors binding sites (TFBSs) were assessed in silico. Bioinformatic functional assessment was conducted using PROMO (ALGGEN) software, which is using data from TRANSFAC database V8.3^25,26^. FASTA sequences for the studied variants were extracted from Ensembl release 98 (http://www.ensembl.org/index.html)^27^. Identification of TFBSs was determined in concordance with the following criteria: human species, all sites and factors.

## Results

### Genotypes frequencies for studied SNPs

For all the investigated polymorphisms, rs1047972 (*AURKA*), rs2273535 (*AURKA*), rs8173 (*AURKA*), rs911160 (*AURKA*), rs2289590 (*AURKB*), rs2241909 (*AURKB*), rs11084490 (*AURKC*), rs758099 (*AURKC*) and rs42873 (*PLK1*) was determined to be in Hardy–Weinberg equilibrium (HWE) in both, case and control groups (*P* > 0.05) (Table 3). After Chi-square test and Fisher exact test were performed to calculate distribution at genotype level (results summarized in Table 3), a significant difference in genotypes frequencies between GBM patients and controls for rs2289590 in *AURKB* (*P* = 0.038) was detected.

**Table 3:** Genotypes frequencies and Hardy–Weinberg equilibrium for the studied polymorphisms.

Genotypes	Control group			Glioblastoma multiforme patients				
	N (%)	HWE		N (%)	HWE		GBM^a^	
		χ^2^	*P* value		χ^2^	*P* value	χ^2^	*P* value
**rs1047972**	202	0.152	0.696	129	1.043	0.307	0.562	0.755
CC	148 (73.3)			92 (71.3)				
CT	49 (24.2)			32 (24.8)				
TT	5 (2.5)			5 (3.9)				
**rs2273535**	203	0.867	0.351	127	2.359	0.124	2.966	0.227
AA	120 (59.1)			66 (52.0)				
AT	69 (34.0)			46 (36.2)				
TT	14 (6.9)			15 (11.8)				
**rs8173**	200	0.017	0.895	127	0.635	0.425	1.955	0.376
CC	97 (48.5)			55 (43.3)				
CG	84 (42.0)			54 (42.5)				
GG	19 (9.5)			18 (14.2)				
**rs911160**	201	0.349	0.554	128	0.031	0.859	0.509	0.755
GG	107 (53.2)			63 (49.2)				
CG	77 (38.3)			53 (41.4)				
CC	17 (8.5)			12 (9.4)				
**rs2289590**	200	3.523	0.060	128	2.275	0.131	**6.548**^b^	**0.038**
AA	62 (31.0)			54 (42.2)				
AC	110 (55.0)			52 (40.6)				
CC	28 (14.0)			22 (17.2)				
**rs2241909**	203	1.186	0.276	128	3.795	0.051	4.809	0.090
AA	87 (42.9)			62 (48.4)				
AG	97 (47.8)			47 (36.8)				
GG	19 (9.3)			19 (14.8)				
**rs11084490**	201	0.0009	0.975	121	0.676	0.410	5.207	0.074
CC	121 (60.2)			88 (72.7)				
CG	70 (34.8)			29 (24.0)				
GG	10 (5.0)			4 (3.3)				
**rs758099**	203	2.107	0.146	128	0.139	0.709	1.752	0.416
CC	103 (50.8)			66 (51.6)				
CT	77 (37.9)			53 (41.4)				
TT	23 (11.3)			9 (7.0)				
**rs42873**	201	0.272	0.601	128	0.111	0.738	0.174	0.917
GG	127 (63.2)			78 (61.0)				
CG	64 (31.8)			43 (33.6)				
CC	10 (5.0)			7 (5.4)				

*GBM* glioblastoma multiforme, *HWE* Hardy–Weinberg equilibrium, *χ*^2^ Chi-square statistics. ^a^χ^2^ analysis between GBM patients and controls. ^b^Fisher statistics. Statistically significant values are highlighted in bold characters (*P* ≤ 0.05).

### Impact of polymorphisms on glioblastoma multiforme risk

Patients with rs2289590 (*AURKB*) heterozygous AC genotype had a lower risk of glioblastoma multiforme (GBM) development in comparison with the reference AA genotype (OR = 0.54, 95% CI = 0.33–0.88, *P* = 0.015) (Table 4). Furthermore, the rs11084490 (*AURKC*) CG genotype was also associated with a decreased GBM risk in comparison with the reference CC genotype (OR = 0.57, 95% CI = 0.34–0.95, *P* = 0.031).

**Table 4:** Risk of glioblastoma multiforme associated with the studied genetic variants.

Genotypes	Glioblastoma multiforme patients	
	OR (95% CI)	*P* value
**rs1047972**		
CC	1 (ref)	
CT	1.05 (0.62–1.76)	0.851
TT	1.60 (0.45–5.70)	0.462
**rs2273535**		
AA	1 (ref)	
AT	1.21 (0.75–1.95)	0.431
TT	1.94 (0.88–4.28)	0.097
**rs8173**		
CC	1 (ref)	
CG	1.13 (0.70–1.82)	0.605
GG	1.67 (0.81–3.44)	0.165
**rs911160**		
GG	1 (ref)	
CG	1.16 (0.73–1.86)	0.513
CC	1.19 (0.53–2.67)	0.658
**rs2289590**		
AA	1 (ref)	
AC	0.54 (0.33–0.88)	**0.015**
CC	0.90 (0.46–1.75)	0.762
**rs2241909**		
AA	1 (ref)	
AG	0.68 (0.42–1.09)	0.113
GG	1.40 (0.68–2.86)	0.353
**rs11084490**		
CC	1 (ref)	
CG	0.57 (0.34–0.95)	**0.031**
GG	0.55 (0.16–1.81)	0.325
**rs758099**		
CC	1 (ref)	
CT	1.07 (0.67–1.71)	0.764
TT	0.61 (0.26–1.40)	0.244
**rs42873**		
GG	1 (ref)	
CG	1.09 (0.67–1.76)	0.713
CC	1.14 (0.41–3.11)	0.799

*OR* odds ratio, *CI* confidence interval, *Ref* reference homozygote. ORs, 95% CIs and *P* values were obtained by multinomial logistic regression analysis. Statistically significant values are highlighted in bold characters (*P* ≤ 0.05).

On the other side, no significant effects on GBM susceptibility were revealed for rs1047972, rs2273535, rs8173 and rs911160 in *AURKA*, rs2241909 in *AURKB*, rs758099 in *AURKC* and rs42873 in *PLK1* (*P* > 0.05).

### Haplotype analysis

**Figure 3:**
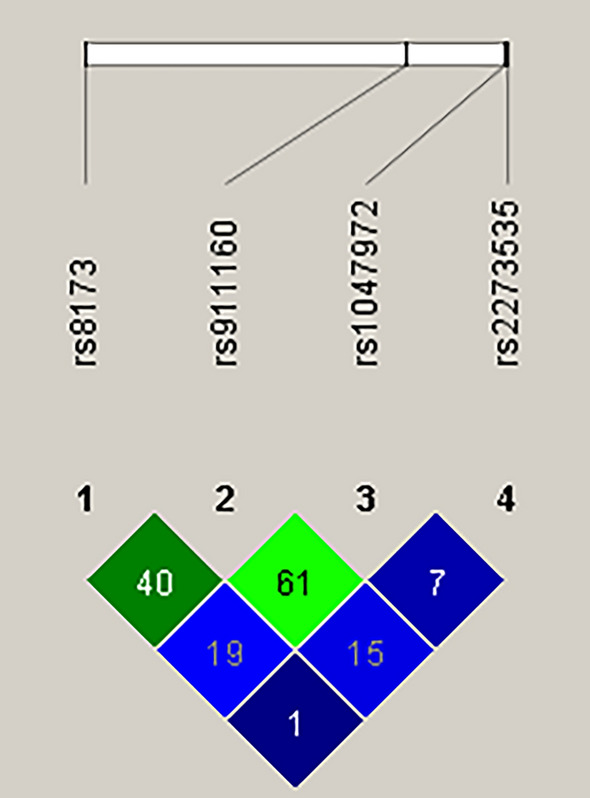
Linkage disequilibrium among single nucleotide polymorphisms in the *AURKA* gene. The color plot represents Lewontin's D′ values and logarithm of odds (LOD). Blue squares, LOD < 2 and D′ < 1; Green squares, LOD ≥ 2 and D′ < 1. The values within the squares refer to the Lewontin's D′ × 100.

After collecting raw genotyping data for the investigated SNPs in *AURKA*, namely rs1047972, rs2273535, rs8173 and rs911160, we carried out haplotype analysis using the Haploview software. The outcome of this analysis revealed that no haplotype block was created with an average Lewontin’s D < 0.8 (Fig. 3), therefore no haplotypes were accessible for the examination of their potential association with GBM risk.

### Bioinformatic analysis of the polymorphisms

In silico analysis revealed that polymorphic sequences in transcription factors binding sites (TFBSs), within non-coding and untranslated regions (UTRs) of *AURKA*, *AURKB*, *AURKC* and *PLK1* genes, bind various transcription factors (TFs). Our results showed that the region comprising G allele of rs911160 (*AURKA*) was linked with C/EBPalpha, C/EBPbeta and GR-beta proteins, while for the C allele, extra binding sites for NF-Y, NFI-CTF and NF-1 were recognized (Table 5). As for rs2289590 (*AURKB*), an additional motif for YY1 binding was identified when C allele was taken into account. In the case of rs11084490 (*AURKC*), there were no observed differences in transcription factor binding site motif (XBP-1), when different alleles, either C or G, were present. The region including C allele of rs758099 (*AURKC*) was related with binding sites for NF-1, NF-Y, XBP-1, ENKTF-1, CTF, PEA3 and POU2F1, while for the region surrounding T allele, NF-1, NF-Y, GATA-1 and TFII-I transcription factors were detected. For the polymorphic sequence which include the G allele of rs42873 (*PLK1*) was demonstrated to be linked with an additional recognition motif for c-Jun DNA-binding factor.

**Table 5:** Bioinformatic analysis of the studied genetic variants.

SNP (gene)	rs911160 (*AURKA)*		rs2289590 (*AURKB)*		rs11084490 (*AURKC)*		rs758099 (*AURKC)*		rs42873 (*PLK1)*	
Alleles	G	C	C	A	C	G	C	T	G	C
Transcription factors^a^	C/EBPalpha	C/EBPalpha	PEA3	PEA3	XBP-1	XBP-1	NF-1	NF-1	GR-alpha	GR-alpha
	C/EBPbeta	C/EBPbeta	TFII-I	TFII-I			NF-Y	NF-Y	AP-2alphaA	AP-2alphaA
	GR-beta	GR-beta	**YY1**				**ENKTF-1**	**TFII-I**	T3R-beta1	T3R-beta1
		**NF-Y**					**XBP-1**	**GATA-1**	**c-Jun**	
		**NF-1**					**CTF**			
		**NFI-CTF**					**POU2F1**			
							**PEA3**			

*SNP* single nucleotide polymorphism.

^a^ Transcription factors binding sites were evaluated using PROMO (ALLGEN) software.

Different transcription factor binding motifs identified for polymorphic alleles of the studied polymorphisms are indicated in bold letters.

## Discussion

Our study focused on the assessment of an association between polymorphisms rs1047972, rs2273535, rs8173 and rs911160 (*AURKA*), rs2289590 and rs2241909 (*AURKB*), rs11084490 and rs758099 (*AURKC*) and rs42873 (*PLK1*), and a risk of glioblastoma multiforme (GBM) development in the population of Bosnia and Herzegovina.

Aurora kinase B (*AURKB*) is a part of chromosomal passenger complex (CPC), which covers processes such as the segregation of chromatids, cytokinesis and histone modifications^28^ and for which has been proven to be overexpressed in different types of cancers including brain, prostate and thyroid^29^. Furthermore, it has been suggested that aurora B overexpression induces abnormalities in chromosome segregation, aneuploidy and tumor development^30^. We examined the rs2289590 polymorphism in *AURKB*, and after Chi-square and Fisher exact tests were performed, a significant difference in genotypes frequencies between GBM patients and control group was observed. Additionally, a protective role of the rs2289590 AC genotype against higher GBM risk was found. In silico analysis of rs2289590 polymorphic region detected additional binding site for the Yin-Yang 1 (YY1) transcription factor, in the presence of C allele.

The YY1 transcription factor is implicated in the regulation of basic processes such as development, cell growth and differentiation, cell cycle progression and apoptosis whereby, it has been demonstrated that YY1 overexpression is linked to an uncontrolled cell proliferation, resistance to apoptotic stimuli and metastasis, thus influencing the process of carcinogenesis itself^31,32^. Transcription factors (TFs) are crucial gene regulators with unique roles during the cell cycle and when their expression is impaired, they fail to provide accurate cellular functioning and stability, which could lead to neoplastic transformation^32,33^. Single nucleotide polymorphisms (SNPs) in regulatory domains can disturb gene expression profile through potential disruption of sequence specific DNA-binding motifs (removing existing and/or creating new ones), therefore altering the binding of correct TFs^34,35^. Moreover, it has been suggested that introns, especially long ones, carrying more functional cis-acting elements could accommodate several TFs binding sites, and consequently affect transcription regulation^36^. Our results for the rs2289590 intron variant in *AURKB* suggested that binding of an extra YY1 transcription factor when C allele is present, could alter *AURKB* expression, which might result in lower susceptibility to GBM occurrence. Roles of introns in transcription regulation have been reported in cell cycle and apoptotic genes, emphasizing the significance of intronic genetic variants in carcinogenesis^37^. In addition to this, SNPs in introns can be used as molecular markers for disease susceptibility and/or as targets in the development of new therapeutics^38^.

Aurora kinase C (*AURKC*) is a member of a chromosomal passenger complex, similarly as Aurora kinase B, which plays important role in mitotic events, segregation and centrosome functioning during meiotic events^13,14^. In cancer cells, the subcellular localization of AURKC is the same as that of AURKB suggesting that they could have similar functions^39^. *AURKC* overexpression has been observed in malignant thyroid cell lines and tissues^40^. Further, it has been demonstrated that AURKC overexpression stimulate centrosome amplification, multinucleation and that its aberrant expression in somatic cells has an oncogenic potential^41^. In this study, we assessed potential relationship between rs11084490 in *AURKC* and GBM risk. A link between heterozygous CG genotype and decreased GBM risk was observed. Polymorphism rs11084490 is located within the *AURKC* 5′ untranslated region. Untranslated regions (UTRs) play role in posttranscriptional regulation of gene expression by a modulation of mRNA stability, nucleo-cytoplasmatic transport, subcellular localization and translation efficiency, thus are involved in fine control of protein product and may affect the quantity and quality of the protein encoded^42^. Several eukaryotic 5′UTR elements/structures, such as RNA G-quadruplexes (RG4s), hairpins, upstream open reading frames (uORFs) and start codons, Kozak sequences around the initiation codons, iron responsive elements (IREs) and internal ribosome entry sites (IRESs), highly affect mRNA translation^43^. It has been shown that 5′ uORF-altering SNPs and mutations, by disrupting motifs within 5′UTR alter downstream protein expression, thus are capable of causing modified effects in terms of susceptibility to certain diseases such as esophageal cancer, multiple myeloma and many others^44,45^. Hence, observed association of the rs11084490 (*AURKC*) polymorphism with a decreased GBM risk in our study, could be due to altered *AURKC* translation mediated by heterozygous (CG) genotype affecting some of the above-mentioned functional motifs in *AURKC* 5′UTR.

## Conclusion

The results of the present study demonstrated that *AURKB* (rs2289590) and *AURKC* (rs11084490) polymorphisms reduce the risk of glioblastoma multiforme development. These findings undoubtedly indicate the existence of the possible positive roles of genetic variations in *AURKB* and *AURKC* genes during brain carcinogenesis. Our data could be beneficial to the future assessments of the functional impact of these polymorphisms. However, our study is based on a reduced number of cases which in a way represents its limitation, and it is therefore necessary that larger prospective studies confirm these allegations.
